# Design and Development of a Telerehabilitation Platform for Patients With Phantom Limb Pain: A User-Centered Approach

**DOI:** 10.2196/rehab.6761

**Published:** 2017-02-15

**Authors:** Andreas Rothgangel, Susy Braun, Rob Smeets, Anna Beurskens

**Affiliations:** ^1^ Research Centre for Autonomy and Participation of People with a Chronic Illness Faculty of Health Zuyd University of Applied Sciences Heerlen Heerlen Netherlands; ^2^ CAPHRI School for Public Health and Primary Care Maastricht University Maastricht Netherlands; ^3^ Kaasa health Duesseldorf Germany; ^4^ Libra Rehabilitation and Audiology Eindhoven/Weert Netherlands

**Keywords:** telerehabilitation, telemedicine, self care, software design, phantom limb, imagery (psychotherapy)

## Abstract

**Background:**

Phantom limb pain is a frequent and persistent problem following amputation. Achieving sustainable favorable effects on phantom limb pain requires therapeutic interventions such as mirror therapy that target maladaptive neuroplastic changes in the central nervous system. Unfortunately, patients’ adherence to unsupervised exercises is generally poor and there is a need for effective strategies such as telerehabilitation to support long-term self-management of patients with phantom limb pain.

**Objective:**

The main aim of this study was to describe the user-centered approach that guided the design and development of a telerehabilitation platform for patients with phantom limb pain. We addressed 3 research questions: (1) Which requirements are defined by patients and therapists for the content and functions of a telerehabilitation platform and how can these requirements be prioritized to develop a first prototype of the platform? (2) How can the user interface of the telerehabilitation platform be designed so as to match the predefined critical user requirements and how can this interface be translated into a medium-fidelity prototype of the platform? (3) How do patients with phantom limb pain and their treating therapists judge the usability of the medium-fidelity prototype of the telerehabilitation platform in routine care and how can the platform be redesigned based on their feedback to achieve a high-fidelity prototype?

**Methods:**

The telerehabilitation platform was developed using an iterative user-centered design process. In the first phase, a questionnaire followed by a semistructured interview was used to identify the user requirements of both the patients and their physical and occupational therapists, which were then prioritized using a decision matrix. The second phase involved designing the interface of the telerehabilitation platform using design sketches, wireframes, and interface mock-ups to develop a low-fidelity prototype. Heuristic evaluation resulted in a medium-fidelity prototype whose usability was tested in routine care in the final phase, leading to the development of a high-fidelity prototype.

**Results:**

A total of 7 categories of patient requirements were identified: monitoring, exercise programs, communication, settings, background information, log-in, and general requirements. One additional category emerged for therapists: patient management. Based on these requirements, patient and therapist interfaces for the telerehabilitation platform were developed and redesigned by the software development team in an iterative process, addressing the usability problems that were reported by the users during 4 weeks of field testing in routine care.

**Conclusions:**

Our findings underline the importance of involving the users and other stakeholders early and continuously in an iterative design process, as well as the need for clear criteria to identify critical user requirements. A decision matrix is presented that incorporates the views of various stakeholders in systematically rating and prioritizing user requirements. The findings and lessons learned might help health care providers, researchers, software designers, and other stakeholders in designing and evaluating new teletreatments, and hopefully increase the likelihood of user acceptance.

## Introduction

Phantom limb pain is a frequent and persistent problem following amputation. Despite many pharmacological and nonpharmacological interventions, up to 80% of patients still suffer from phantom limb pain many years after the amputation [1-3]. According to a recent trial [3], 63% of a sample of 3234 amputees with an average time since amputation of 33 years, were still suffering from phantom limb pain. These data illustrate the chronic nature of this disorder, which is accompanied and maintained by a wide range of changes in the peripheral [4] and central nervous system [5]. Achieving sustainable favorable effects on phantom limb pain requires therapeutic interventions such as mirror therapy [6] that target these maladaptive neuroplastic changes in the central nervous system.

Two recent systematic reviews [7,8] reported that despite the potential merits of mirror therapy, the quality of evidence for patients with phantom limb pain is still low and a detailed description of how to deliver the intervention is lacking. Therefore, we recently developed an evidence-based clinical framework for mirror therapy for patients with phantom limb pain [9] that is currently being tested for effectiveness in a multicenter randomized controlled trial [10]. Given the chronic nature of phantom limb pain, continuous training with at least one session a day over a period of several weeks to months seems to be needed to achieve sustainable treatment effects [7]. However, resources in clinical practice are generally scarce, which necessitates unsupervised training by patients to achieve the desired training intensity. Unfortunately, patients’ adherence to unsupervised training is generally poor [11], implying the need for effective strategies to support long-term self-management by patients with phantom limb pain.

One possible strategy might be the use of information and communication technology such as telerehabilitation, which allows patients to continue their treatment program independently at their own homes. Furthermore, therapists can create tailored exercise programs, improve their guidance for self-administered exercises, and monitor phantom limb pain. Problems that occur during self-management can be discussed with the supervising therapist and the treatment program can be modified according to patient’s preferences to increase long-term adherence to self-administered exercises [12,13]. The use of telerehabilitation has been shown to enhance treatment intensity [14], self-efficacy [15,16], and compliance with self-administered exercises, that in turn correlates positively with the effects of the intervention [17]. Moreover, the implementation of these potential time- and cost-saving strategies might lead to increased accessibility and enhanced continuity of care [18]. Data regarding the effects of telerehabilitation in patients with phantom limb pain is sparse. In a recent study [19], a teletreatment for 2 patients with phantom limb pain using mirror therapy was described. This teletreatment solely consisted of email instructions by a physician on how to deliver self-administered mirror therapy. Both the patients reported complete recovery from phantom limb pain after daily exercises for 4 and 8 weeks, respectively. However, the teletreatment was restricted to email instructions, and it remains unclear as to how the content of the teletreatment was developed and whether the end users were involved during the design of the system.

To facilitate user acceptance, such teletreatments have to be easy to use [20], match the requirements and preferences of the end users [21], and fit in their personal context [22]. This is supported by theoretical models such as the technology acceptance model (TAM) [23,24] and the unified theory of acceptance and use of technology (UTAUT) [25,26] that assume that user acceptance and the intention to use a telemedicine service is predicted by factors such as perceived usefulness, perceived ease of use, as well as intrinsic motivation and social influence. Therefore, it is essential to involve the end users in the design and development of any new telerehabilitation platform. In the PAtient Centered Telerehabilitation (PACT) project [10], we developed an innovative mobile telerehabilitation platform using mirror therapy for patients with phantom limb pain following lower limb amputation. Patients and physical and occupational therapists were involved throughout the entire platform development process.

The aim of this study was to describe the user-centered approach that guided the design and development of the telerehabilitation platform.

The following research questions were addressed:

Which requirements are defined by patients with phantom limb pain following lower limb amputation and the occupational and physical therapists treating these patients regarding the content and functions of a telerehabilitation platform, and how can these requirements be prioritized to develop a first prototype of the platform?

How can the user interface of the telerehabilitation platform be designed so as to match the predefined critical user requirements, and how can this interface be translated into a medium-fidelity prototype of the platform?

How do patients with phantom limb pain and their treating therapists judge the usability of the medium-fidelity prototype of the telerehabilitation platform in routine care, and how can the platform be redesigned based on their feedback to achieve a high-fidelity prototype?

Our description of this process and the lessons learned along the way aims to offer insights into the complexity of the user-centered design process and illustrates the necessity to address the needs of different stakeholders to achieve a platform that is easy to use and fits in with the daily routines of the users. Our findings might help health care providers, researchers, software designers, and other stakeholders in designing and evaluating new teletreatments.

## Methods

### Study Design

**Figure 1:**
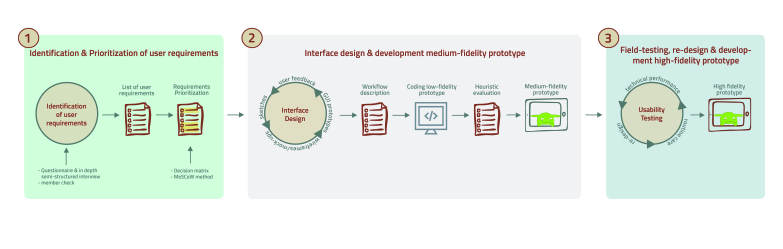
Overview of the 3 phases and methods used throughout the user-centered approach.

The framework to improve the uptake and impact of eHealth technologies [27] and the method of agile software development [28] were used in an iterative user-centered design process to develop the telerehabilitation platform in 3 phases (Figure 1).

Important topics that are mentioned in the framework of van Gemert-Pijnen [27] such as a participatory development and design approach, value specification through identification of user requirements, as well as persuasive design techniques and continuous evaluation cycles were also addressed in this study.

### Recruitment of Patients

We used purposive sampling to achieve a wide range of patient characteristics (eg, age, gender, reason for amputation, time since amputation) to obtain a rich data collection. The principal investigator (AR) identified eligible patients by contacting patient support groups and orthopaedic technicians and placing Web-based advertisements in Germany. In addition, the therapists who participated in the interviews selected patients whom they had treated in the past or whom they were currently treating. Adult patients with unilateral amputation of the lower limb and sufficient cognitive and linguistic capacities to participate in a 1-hour interview were included. In addition, patients needed to have sufficient experience in using mirror therapy, which was defined as having attended at least five treatment sessions during the past 12 months. Selection of patients was based on the judgment of the recruiting principal investigator or therapists.

### Recruitment of Therapists

The principal investigator identified physical and occupational therapists by email or phone via existing networks in Germany. The professionals needed to have sufficient experience in using mirror therapy for patients with phantom limb pain, which was defined as having treated at least three patients during the past 12 months. Again, we tried to include a wide range of therapist characteristics (eg, profession, age, experience, work setting) to obtain a rich data collection.

#### Phase 1: Identification and Prioritization of User Requirements (Research Question 1)

In the first phase, a questionnaire followed by a semistructured interview was used to identify the user requirements of both the patients suffering from phantom limb pain and the physical and occupational therapists. The reported requirements were then prioritized using a decision matrix.

##### Collection and Analysis of Data

We developed a structured questionnaire for patients and therapists that contained questions on patient and therapist characteristics such as level and side of amputation, a case description of a patient with phantom limb pain to illustrate the principle of telerehabilitation, and 3 general items regarding the content and functions of the platform (eg, ‟which information, content or functions should be included in the telerehabilitation platform enabling tailored support of your patients regarding self-delivered exercises?”). In addition, 3 therapist respectively 6 patient questions regarding user acceptance, barriers and facilitators, and context of use were included (eg, which aspects are relevant to increase patient and therapist acceptance of the telerehabilitation platform?). The questionnaire was checked on integrity and comprehensibility by 5 therapists and 1 patient representative. After some minor text revisions and after participants gave informed consent, the principal investigator sent the questionnaire by email to all patients and therapists who were to participate in the interviews 2 weeks before the interview took place. The completed questionnaire was to be returned at least one day before the interview. The principal investigator checked the data regarding the telerehabilitation platform before the interview took place to prepare for the interview and refined in-depth questions on the various topics.

All interviews were conducted by the principal investigator in a quiet room at the patient’s home or at the professional’s clinic. The interviews lasted approximately 1 hour and were digitally audio-taped and subsequently transcribed using the f4 software (audiotranskription, Marburg, Germany). In addition, the principal investigator took field notes after each interview describing the context of the interview. After 6 interviews had been transcribed, the principal investigator used data analysis to check which topics emerged, and recruited additional patients and therapists until data saturation was achieved.

The data regarding patient and therapist characteristics were extracted from the questionnaires and displayed in a frequency table. Data regarding the topics relating to the telerehabilitation platform were analyzed using directed content analysis [29]. The initial coding scheme was based on the topics of the questionnaire. This scheme was extended as new topics emerged from the data analysis. After each interview, the data were summarized by topic in a table and were subsequently sent to the interviewee, who was asked to check the data for integrity and correctness (member check). The interviewees returned the adjusted summary of the data to the principal investigator by email. A sample of 2 patient and 2 therapist interviews was independently analyzed by another researcher (SB) and the results were discussed with the principal investigator to reach consensus about the data analysis. Finally, all data from the interviews were clustered into topics and the user requirements regarding each topic were specified in a table to create a requirements catalog.

##### Requirements Prioritization

The user requirements were subsequently prioritized to decide which requirements from the requirements catalog were critical to include in the first prototype of the telerehabilitation platform. We developed a decision matrix incorporating 3 different criteria to reflect the views of various stakeholders in the project (patients, therapists, researchers, and software development team, see also Table 2):

Best available evidence: A systematic literature review regarding the clinical framework of mirror therapy for patients with phantom limb pain was conducted in a preliminary stage [9]. Literature was screened to identify studies supporting the relevance of each reported user requirement.

Technical complexity: Members of the software development team were also asked to rate the different requirements in order to determine the technical complexity of each requirement. They were asked whether implementation of each requirement would be time-consuming or expensive. The technical complexity of each requirement was assessed by 3 engineers from the software development team (Kaasa health, Duesseldorf, Germany) using an 11-point numeric rating scale (0=very low, 10= very high complexity).

Importance of requirements: The importance of the requirement was primarily defined by the number of respondents who mentioned the requirement and whether or not there was agreement between patients and therapists (eg, the more respondents mentioned the same requirement, the more important the requirement). However, an exception was made for requirements that were only mentioned by a minority of users but were nevertheless regarded as important by the research team that rated the priority of requirements.

Based on these criteria, 3 members of the research team (RS, AJB, AR) rated the priority of each user requirement independently on a 4-point numeric rating scale according to the MoSCoW prioritization method (1=Must have, 2=Should have, 3=Could have, 4=Won’t have at this time) [30].

Only requirements that were scored as priority stage 1 or 2 by at least two of the 3 raters were defined as critical for the first prototype of the telerehabilitation platform.

#### Phase 2: Interface Design and Development of Medium-Fidelity Prototype (Research Question 2)

Based on the critical user requirements defined in phase 1, the interface of the telerehabilitation platform was designed using design sketches, wireframes, and interface mock-ups (Balsamiq Mockups, version 2.2.10, Balsamiq Studios, Sacramento). All critical user requirements belonging to 1 specific category were used to build the first design sketches incorporating these requirements. In the next step the interface designer of the software development team converted these mock-ups into graphical user interface (GUI) prototypes. The GUI prototypes were shown in several iterative phases, on screen or paper, to a sample of 6 patients and 5 therapists who had been interviewed in phase 1, to provide feedback regarding the content and design of the prototypes. Their feedback was summarized and discussed with the interface designer, to refine the GUI prototypes. Evaluation of GUI prototypes continued until the majority (>50%) of patients and therapists made no further comments, and the final interface design emerged. For each category of user requirements, a workflow description was composed in which the final GUI was used to illustrate the sequential steps to be taken by the users when operating the application. Based on this workflow description, the source code was programed for each application to develop a low-fidelity prototype of the telerehabilitation platform.

##### Heuristic Evaluation

The usability of the low-fidelity prototype was tested in a laboratory situation by 3 therapists who had already been involved in phase 1, as well as 10 physical therapy students and 4 evaluators from the software development team, using the criteria of Nielsen [31]. Typical user tasks such as logging in and recording a pain score or selecting a tailored exercise program were developed, to enable the evaluators to rate the prototype in terms of existing usability principles (‟heuristics”). We developed a criteria matrix (Table 2) in which each evaluator noted their feedback on each heuristic. Subsequently, the severity of each usability problem was rated on a 5-point numeric scale (1= I don’t agree that this is a usability problem at all, 5=Usability catastrophe) according to the frequency and persistence of the usability problem and its impact on the workflow [32]. The results of the heuristic evaluation were reported to the software development team, who fixed usability problems with a minimal severity score of 3 to create a medium-fidelity prototype of the telerehabilitation platform.

#### Phase 3: Field-Testing in Routine Care, Redesign and Development of High-Fidelity Prototype (Research Question 3)

Following the heuristic evaluation, the medium-fidelity prototype was tested for usability and technical performance in routine care by 2 physical and 3 occupational therapists who had already taken part in phase 1 and also participated in the multicenter trial [10]. Each therapist was asked to select 2 patients with phantom limb pain whom they were currently treating. The participating therapists were trained regarding the content and application of the telerehabilitation platform. Subsequently, each therapist was asked to instruct patients with phantom limb pain on how to use the telerehabilitation platform before patients were discharged from the rehabilitation center. After discharge, patients and therapists used the telerehabilitation platform for a period of 4 weeks. During this period, the users were encouraged to use various aspects of the telerehabilitation platform (eg, personal communication with patient or therapist or other patients, exercise programs, monitoring of phantom limb pain) and were asked to note any usability problem by means of an in-app feedback system that automatically transferred the user feedback to the software development team. In addition, patients and therapists were phoned once a week by the principal investigator to assess usability problems that were not automatically recorded through the in-app feedback system. All usability problems were listed in a standardized bug log and scored by the principal investigator for priority (low, medium, high). The technical performance of the prototype was evaluated using data logging. The issues mentioned in the bug log were continuously forwarded to the software development team that redesigned the prototype until the users reported no more major bugs and a high-fidelity prototype of the telerehabilitation platform had been achieved.

##### Ethical Approval

This study has been approved by the Ethics Committee of the Medical Faculty of Cologne University, Cologne, Germany (approval no. 12-029).

## Results

### Phase 1: Identification and Prioritization of User Requirements (Research Question 1)

In total, 11 patients (6 female) and 10 therapists (8 female) were recruited for the interviews until data saturation was achieved. The sample of patients was very heterogeneous as shown in Table 1.

**Table 1:** Characteristics of patients participating in the interviews.

Patient	Age (years)	Gender^a^	Work status	Time since amputation (months)	Side of amputation	Level of amputation	Reason for amputation	Information and communications technology experience
1	22	F	Student	15	Left	TT^b^	Trauma	High
2	49	M	Part-time	12	Right	TT	Trauma	Medium
3	56	F	Retired	5	Right	TT	Vascular	Low
4	64	M	Retired	116	Right	HE^c^	Vascular	High
5	49	F	Retired	27	Right	HE	Vascular	High
6	70	M	Retired	36	Left	TF^d^	Vascular	Low
7	39	F	Retired	39	Left	HE	Infection	High
8	49	M	Retired	328	Right	HP^e^	Trauma	High
9	47	M	Retired	35	Right	TF	Vascular	Medium
10	59	F	Full time	3	Right	TF	Vascular	Low
11	24	F	Student	45	Left	F^f^	Trauma	High

^a^F: Female, M: Male.

^b^TT: Transtibial.

^c^HE: Hip exarticulation.

^d^TF: Transfemoral.

^e^HP: Hemipelvectomy.

^f^F: Foot.

The occupational (n=5) and physical (n=5) therapists (age range 23-57 years) had extensive work experience in treating amputees ranging from 5 to 28 years. Three therapists worked in a hospital, 4 in a rehabilitation center and 3 in a private practice. Three therapists reported a low level, 3 reported a medium, and 4 reported a high level of experience in using information and communication technology.

#### Requirements Defined by Patients and Therapists

A total of 63 patient requirements and 64 therapist requirements were identified. After the prioritization process, 24 patient requirements and 35 therapist requirements remained that were classified as critical for the first prototype of the telerehabilitation platform (Table 2). Seven categories of patient requirements were identified: Monitoring (eg, monitoring of phantom pain and self-administered exercises), training programs (eg, mirror therapy, mental practice), communication (eg, text messages, videoconferencing), settings (eg, personal data, reminder), background information (eg, phantom pain, training programs), and log-in and general requirements (eg, privacy, gamification). With respect to the requirements of therapists, 1 additional category emerged: Patient management (eg, creating a new patient, patient overview).

We decided to develop a mobile app of the telerehabilitation platform as the majority of the patients and therapists preferred mobile access to the platform in order to be more flexible regarding the time and place of platform use.

**Table 2:** Prioritization of user requirements using the decision matrix (example shows 4 out of 64 therapist requirements from the category ‟monitoring”).

ID	Category 1: Monitoring	Decision criteria					
	Description of requirement (number of entries)	Literature^a^ (+ or − or ?)	Defined by majority of users^b^ (+ or −)	Consensus patient therapist^c^ (+ or −)	Complexity 0= very low 10= very high	Priority^d^ 1=high 4=low	Notes
1^e^	The system must be able to monitor the intensity of phantom limb pain, so that the therapist is able to evaluate its course over time (10/10)	+ Barbin et al [8] Rothgangel et al [9]	+	+	5	1 1 1	
2	The system has to record the perceived position and range of motion of the phantom limb (1/10)	+ Schmalzl et al [33] Mercier and Sirigu [34] Moseley [35] Sumitani et al [36]	-	-	8	3 4 3	Consider for clinical trial
3^e^	The system must enable the therapist to control the frequency and quality of self-delivered exercises (eg, video recording, text messages) (10/10)	+ Darnall and Li [11] Beaumont et al [37] MacIver et al [38]	+	+	8	1 1 2	Camera of tablet has no wide angle—poor display window
4	The system has to record the perceived difficulty of self-delivered exercises (3/10)	+ Mercier and Sirigu [34] Beaumont et al [37] Giraux and Sirigu [39]	-	-	5	3 2 3	

^a^+= yes, −= no, ?=unclear.

^b^+=Requirement defined by >50% of users.

^c^+=consensus between at least one patient and one therapist.

^d^1=must have, 2=should have, 3=could have, 4=won’t have this time.

^e^Based on the decision criteria and priority rating only requirements with ID 1 and 3 were defined as critical for the first prototype.

### Phase 2: Interface Design and Development of Medium-Fidelity Prototype (Research Question 2)

Based on the 7 categories of user requirements identified, a mobile app was developed for each category, incorporating all user requirements belonging to this category, using an iterative design process. The development process is illustrated in the following section using the example of phantom limb pain monitoring.

**Figure 2:**
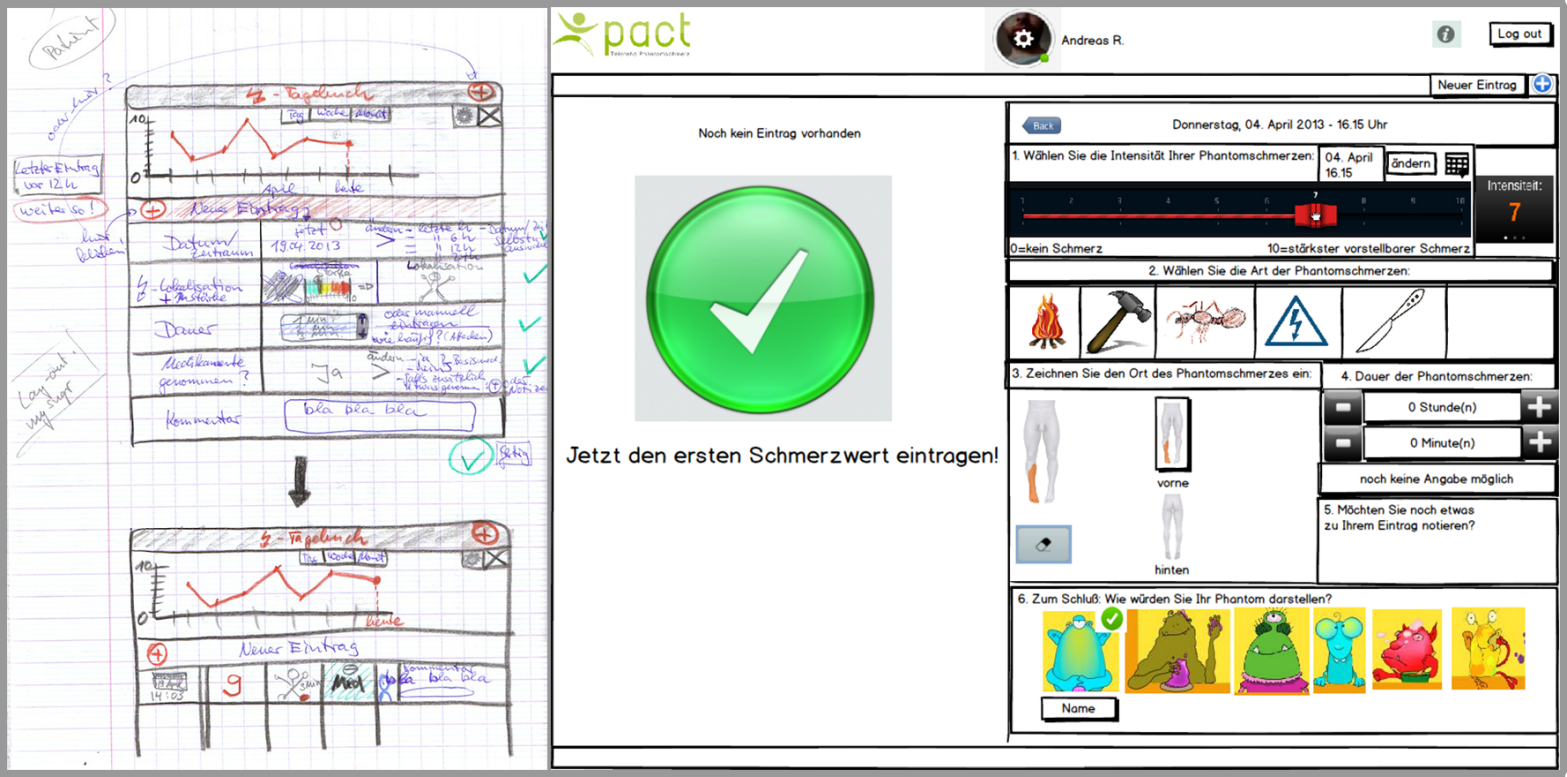
First design sketches and mock-ups of phantom limb pain monitoring.

Ten patients and all therapists agreed that the telerehabilitation platform should be able to monitor the frequency, duration, type, and intensity of phantom limb pain. These aspects were integrated in the first userface design sketches and mock-ups of the mobile app for monitoring of phantom limb pain (Figure 2).

**Figure 3:**
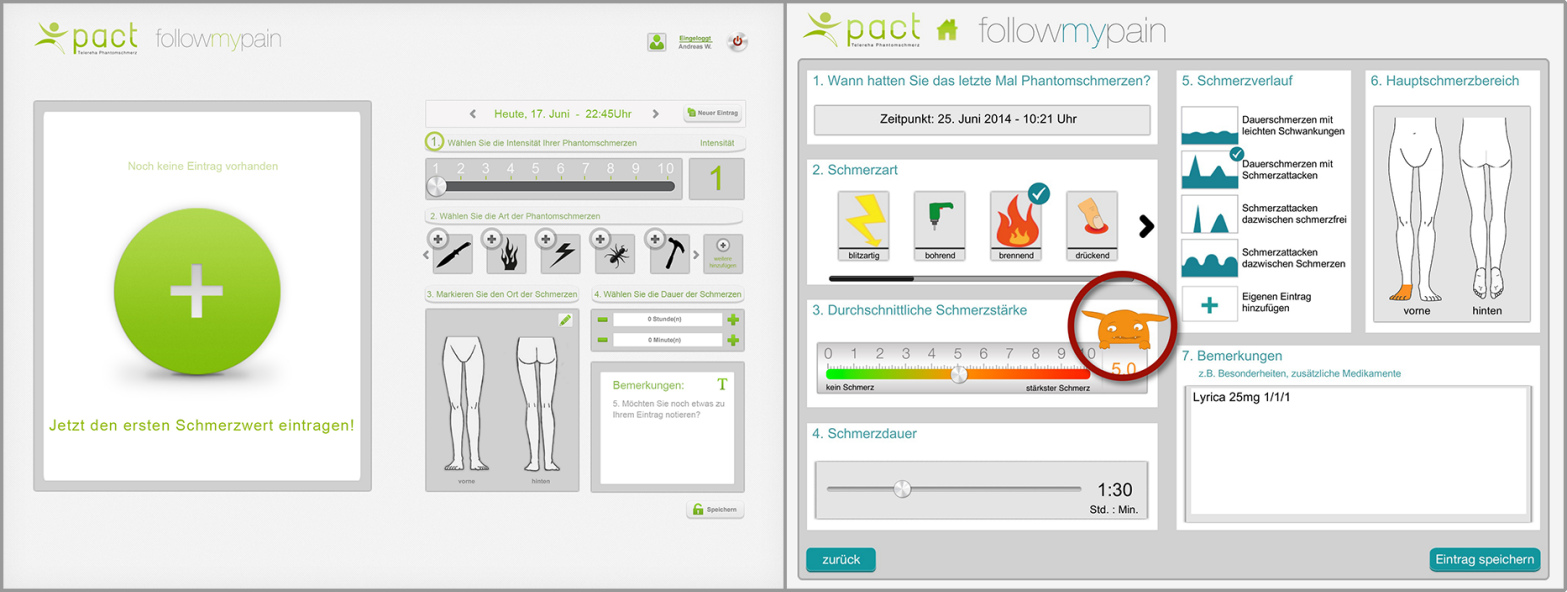
First graphical user interface (GUI) prototype and final interface design of phantom limb pain monitoring after 7 iterative rounds.

These mock-ups resulted in the first graphical user interface (GUI) prototypes (Figure 3). The feedback from patients and therapists regarding the GUI prototypes showed that 6 patients and 5 therapists required a more compact and comprehensive overview of the most important aspects of phantom limb pain. In addition, 7 patients wished to integrate some gaming elements to enliven the use of the application. In response to this, a little monster symbolizing the phantom limb pain was introduced (Figure 3). The final interface design of the mobile app for monitoring phantom limb pain emerged after 7 iterative rounds with patients and therapists.

#### From Low to Medium-Fidelity Prototype

**Figure 4:**
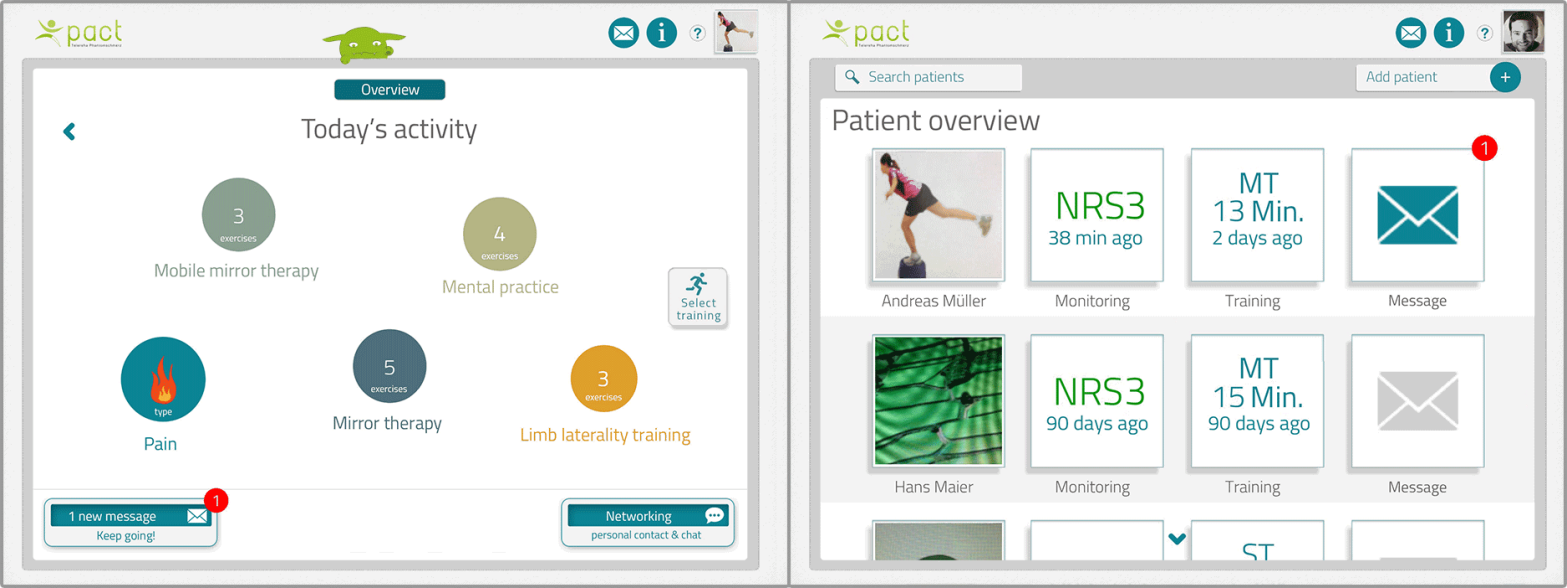
Low-fidelity prototype of patient and therapist interfaces of the telerehabilitation platform.

**Figure 5:**
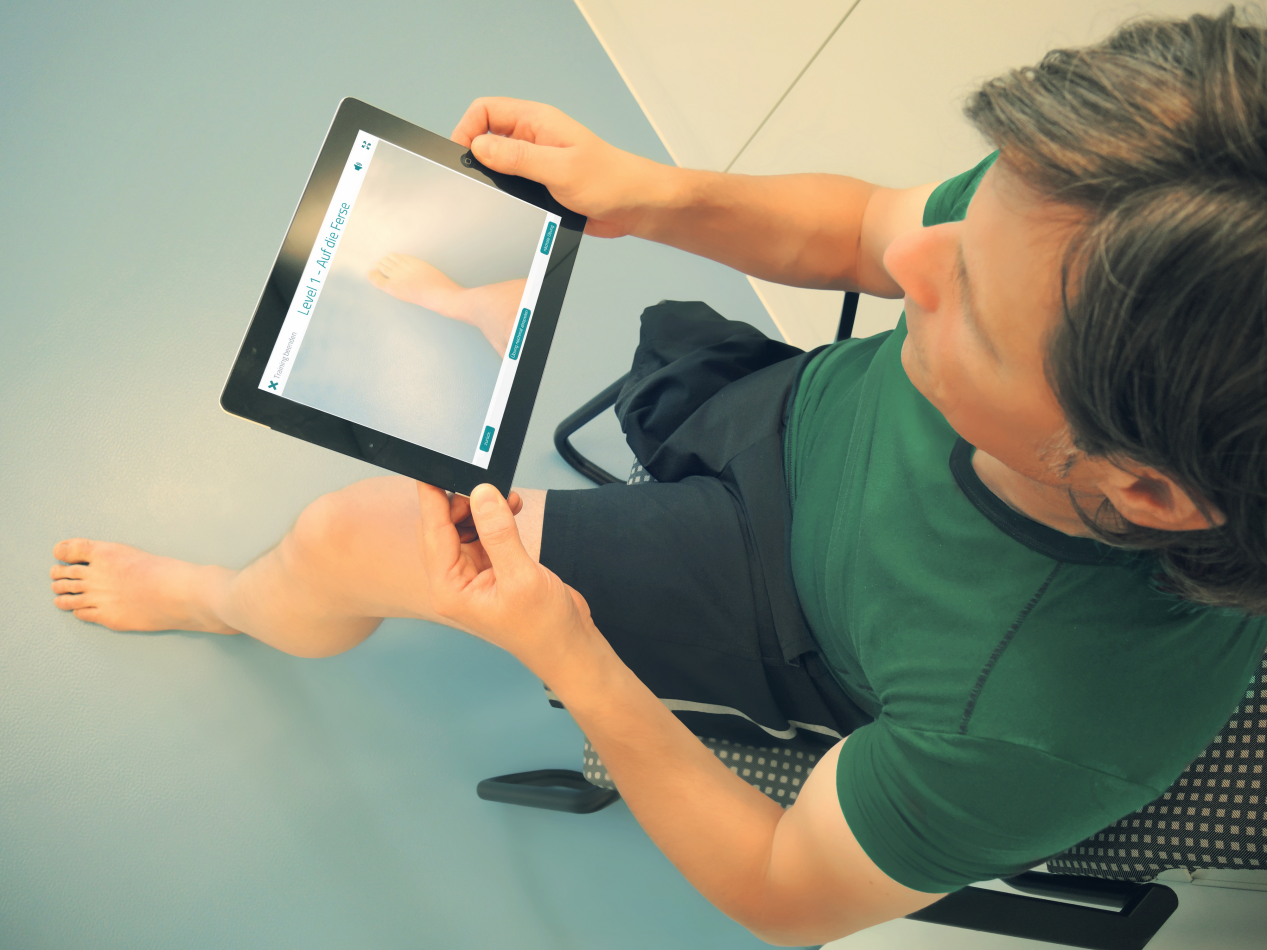
Mobile mirror therapy facilitated by augmented reality using the tablet-integrated camera.

The coding process based on the workflow description resulted in a low-fidelity prototype of 5 different individual applications that were included in the main menu of the patient interface of the telerehabilitation platform (Figure 4): monitoring phantom limb pain, traditional mirror therapy, mobile mirror therapy facilitated by augmented reality using the tablet-integrated camera (Figure 5; Multimedia Appendix 1), mental practice including relaxation exercises and limb laterality recognition training.

The main menu was also coded as 1 individual application and featured additional functions such as an overview of exercise programs and training history, background information, personal settings, or communication with a personal therapist and other patients (eg, short message system, videoconferencing).

The main menu of the therapist interface of the low-fidelity prototype integrated 4 different applications in a coherent overview, to enable easy access for the professional: personal and medical data of patient, monitoring of phantom limb pain and self-administered exercises, creation of individual exercise programs, and communication with individual patients (Figure 4). In addition, the main menu contained personal settings for the therapist and a patient management system with an overview of patients currently being treated by the therapist, as well as options for searching and adding new patients.

#### Heuristic Evaluation

The group of evaluators who rated the usability according to Nielsen criteria identified several usability problems in the low-fidelity prototype, as shown in Table 3. Usability problems were found to occur in different areas of the prototype (eg, log-in, profile settings, exercise programs). For example, the software did not provide sufficient information about the system status during various tasks such as sending messages.

**Table 3:** Results of heuristic evaluation of the low-fidelity prototype (one example per heuristic shown).

Type of heuristic	Description of usability problem	Frequency of problem 0= never 10=very often	Impact on workflow 0= low 10=very high	Persistence low or medium or high	Severity rating 1-5^a^
Visibility of system status	The system provides no feedback about whether a message has successfully been sent or not.	7	5	High	4
Match between system and the real world	If the user takes a profile picture the system shows it upside down.	3	3	Medium	3
User control and freedom	It is not clear where the user can log out.	10	7	Low	4
Consistency and standards	It is not clear whether the phrase video training means the same as the phrase mental practice.	2	0	Low	2
Error prevention	The system does not provide feedback on how to get back to the main menu after the training has been completed.	10	8	Medium	4
Recognition rather than recall	There is no tutorial that guides the user through the different sections of the application.	2	3	High	3-4
Flexibility and efficiency of use	There is no option to skip the instruction videos in the training programs.	10	5	Medium	4
Aesthetic and minimalist design	The text in the video selection frame is redundant as it is a repetition of the title.	8	0	Low	2
Helping users recognize, diagnose, and recover from errors	There is no error message when the Internet connection is timed out or a wrong password is used during log-in.	10	10	Medium	4
Help and documentation	The help icon in the limb laterality recognition training does not work.	2	1	High	2

^a^Severity rating: 1= I don't agree that this is a usability problem at all, 2=Cosmetic problem only: need not be fixed unless extra time is available, 3=Minor usability problem: fixing this should be given low priority, 4=Major usability problem: important to fix, so should be given high priority, 5=Usability catastrophe: imperative to fix this before product can be released.

All usability problems that were rated with a minimal severity score of 3 were fixed by the software development team in order to build a medium-fidelity prototype of the telerehabilitation platform.

### Phase 3: Field Testing in Routine Care, Redesign and Development of High-Fidelity Prototype

During the 4 weeks of field testing of the medium-fidelity prototype in routine care, patients and therapists reported additional usability problems through the in-app messaging system and during the weekly telephone calls regarding the following topics: (1) Problems related to the Internet connection (eg, delayed data transfer and log-in); (2) Messaging system (eg, message is not completely visible in the text fields, no confirmation if the message was successfully sent, message not received by user); (3) Data management (eg, system displays wrong dates and patient scores); (4) Patient management (eg, failure to add new patients and save a tailored exercise program); and (5) Interface design (eg, overlap of text and icons, missing icons).

The software development team continuously redesigned the medium-fidelity prototype. As soon as a new version of the telerehabilitation prototype was available, the software for patients and therapists was updated so they were able to test it in routine care.

#### High-Fidelity Prototype

**Figure 6:**
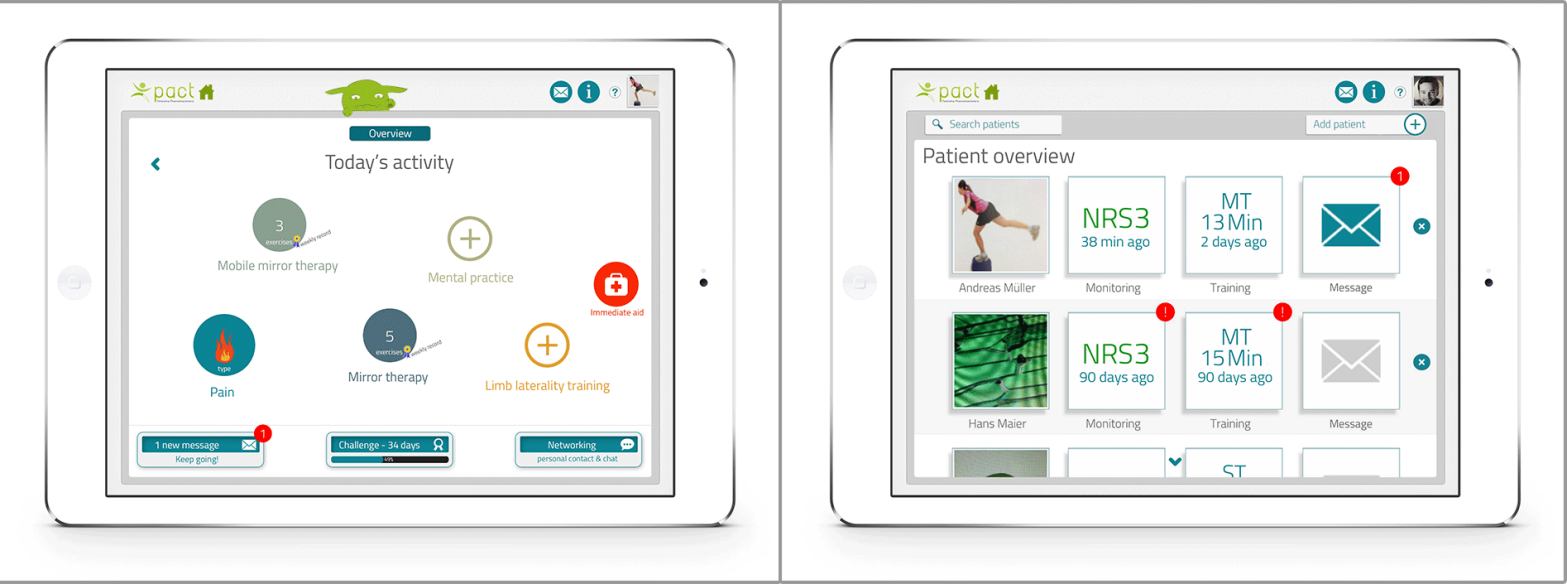
High-fidelity prototype of patient and therapist interfaces of the telerehabilitation platform.

After all major bugs had been fixed, additional graphics such as a home button were added to the patient interface. In addition, some elements to facilitate patient compliance (eg, group challenges using high scores, awards) were incorporated in the high-fidelity prototype (Figure 6). The button to select a training program was replaced by a button ‟immediate action” to enable patients to immediately start mobile mirror therapy in case of an acute attack of phantom limb pain. Tapping on the colored circles starts the individual exercise programs. A new tutorial on how to use the different functions of the platform was also included in the main menu for patients and therapists. A new button to add and delete patients was included in the therapist interface (Figure 6).

## Discussion

In this project, an interdisciplinary software development team consisting of several stakeholders (patients, health care professionals, researchers, and information technology [IT] experts) took part in designing and developing a mobile telerehabilitation platform for patients with phantom limb pain by means of an iterative user-centered design process. Each of the 3 research questions was answered in a separate phase of the process.

### Principal Findings

The first phase of the study aimed to identify the requirements defined by patients and therapists regarding the content and functions of a telerehabilitation platform and how these requirements could be prioritized to develop a first prototype of the platform.

The users defined an extensive list of requirements (N=127) regarding the topics of monitoring, training programs, communication, settings, background information, log-in, general requirements, and patient management. The limited time and budget available meant that not all requirements could be incorporated in the platform. Hence, it was essential to have a decision aid based on clear criteria that enabled systematic prioritization of user requirements and ensured the identification of the most critical requirements to include as a starting point in the first prototype of the telerehabilitation platform. To this end we developed a decision matrix reflecting the views of various stakeholders based on 3 different criteria: best available evidence [9], importance of the requirement, and the technical complexity (time or money) of implementing the requirement in the platform.

The first 2 criteria were clear and straightforward to use. The last criterion, however, required frequent discussion with the software team and turned out to be an important and restricting factor in deciding whether or not a requirement was implemented. Some user requirements such as ‟monitoring the phantom limb pain” were technologically easy to develop and implement, whereas some others, such as ‟perceived position and range of motion of phantom limb” were technologically complex to design. It has to be mentioned that depending on the user characteristics (eg, age, experience in using IT) it was difficult for some users to provide reasonable information regarding the content and functionalities of the platform. For this reason some requirements were only mentioned by 1 or 2 users, nonetheless providing valuable information. In order to also meet the needs that were mentioned by a minority of users, 3 members of the research team that rated the priority of requirements decided whether these requirements provided important information that should be taken into account. Overall, the decision matrix was very helpful and enabled us to systematically rate and prioritize all requirements.

The second phase of the study was used to assess how the user interface of the telerehabilitation platform could be designed to match the critical user requirements and how the interface could best be translated into a medium-fidelity prototype.

It appeared to be crucial to involve the users and other stakeholders early and often in the design process, that is in line with results from a recent scoping review [40]. The potential future users were shown mock-ups and prototypes of graphical user interfaces of the low and medium-fidelity prototypes of the platform, incorporating the predefined user requirements. During this iterative process, the users were able to check whether their requirements had been sufficiently addressed. They highly appreciated the possibility to cocreate the application with the interdisciplinary software team. In particular, participants were enthusiastic about discussing with other users their ideas regarding the functions and interface design, and to see how their feedback was incorporated in the subsequent prototypes. In addition, some functions and interface design issues that were suggested by the software team, such as adding a Facebook sign-in button, were rejected because the users did not consider them relevant. As soon as the final interface design emerged, it was important to provide the software developers with a structured and logical workflow description so that they were able to code a first prototype matching the critical user requirements. However, continuous redesign of the first prototype was required to achieve a medium-fidelity prototype, as several usability problems were identified through heuristic evaluation.

This close cooperation with the users and other stakeholders gave us valuable insights into critical requirements and resulted in a telerehabilitation platform that will most likely fit the main requirements and wishes of the end users.

Phase 3 of the project assessed the usability of the medium-fidelity prototype of the telerehabilitation platform in routine care as judged by patients with phantom limb pain and their treating therapists. This information was necessary to redesign the platform into a high-fidelity prototype.

An important step during the iterative design process was field testing the platform in routine care, which contributed greatly to improving the usability of the platform. During this process the users continuously identified additional problems that had not been detected before through heuristic evaluation. When field testing started, the users rated the usability of the medium-fidelity prototype as poor because of several problems such as delayed data transfer or problems regarding the login process. It was important to discuss the usability problems continuously with the software development team and to regularly provide the users with an improved version of the platform, to gradually increase its usability to achieve a high-fidelity prototype. However, at a certain point in the development process we had to stop improving the platform and start the multicenter trial in order to evaluate the effects of the platform [10]. This time was difficult to set as there are no formal criteria to decide when to stop the prototype design process. Development of the platform stopped after all critical issues had been resolved and time and budget restrictions did not allow any more reported bugs to be addressed, despite the fact that less critical malfunctions kept occurring. The latter implies that in the platform that is currently being evaluated in a multicenter trial [10], there could still be some minor malfunctions which can potentially influence user acceptance.

### Strengths and Limitations

In our experience it is important to take sufficient time for the different stakeholders to get to know and understand each other. It is necessary that the different stakeholders learn to speak each other’s language in order to work effectively together and correctly transform the wishes and requirements of the users into the design of the tool. Even though the involvement of the users and other stakeholders made the process time-consuming, we believe that it is a crucial factor in building an eventually successful and user-friendly platform.

A potential limitation of this study could be that the same sample of patients and therapists (except for the patients who were recruited for usability testing in routine care) was used throughout the development process of the telerehabilitation platform. This enabled patients and therapists to check whether the requirements, which they defined, were sufficiently addressed in the first prototypes of the platform. However, using the same sample also carries the risk that the views of novel users without prior knowledge regarding the platform are insufficiently addressed. This may have resulted in a lower number of reported usability problems. This potential underestimation of usability problems was tackled by including novel patients who were not familiar with the technology during field-testing in routine care.

Patients and therapists who participated in field testing had limited time to practice in using the telerehabilitation platform. However, this time frame seemed appropriate to evaluate the usability and ease of use of the system as it reflected the situation of a first-time user [41]. Field testing does not provide sufficient insights into user compliance with and acceptance of the platform. This will be further analyzed in our multicenter trial [10], in which patients use the telerehabilitation platform over a period of 6 months.

### Comparison With Prior Work

Prioritization of user requirements is still a challenge in software engineering [42]. Recently, it has been recommended that requirements should be prioritized from a user point of view [42]. There are many difficulties in defining which factors should be taken into account when setting the priorities. For example, Moisiadis [43] argues that prioritizing requirements should involve representatives from different stakeholders with a vested interest in the success of the development project. To our knowledge ours is one of the first studies to use a decision matrix incorporating the views of different stakeholders to systematically rate and prioritize user requirements within a telehealth project.

A recent study [19] described a teletreatment for patients with phantom limb pain using mirror therapy. In contrast to our study, this teletreatment consisted solely of email instructions by a physician on how to deliver self-administered mirror therapy. In our experience, however, users have many other requirements regarding the functionalities of a telerehabilitation platform, such as monitoring the phantom limb pain, communication with a personal therapist and other patients, as well as tailored management of the training programs.

In recent years, several telerehabilitation platforms have been developed for different patient groups, such as those with musculoskeletal [44], neurological [45], or pulmonary conditions [46]. However, it remains unclear whether these platforms were developed following a strict user-centered approach. Lack of user acceptance is one of the major barriers to the deployment of services in many telehealth projects [47,48], mainly because relevant user preferences and usability issues have not been taken into account [41]. Early and frequent involvement of end users in the design process, as presented in this study, could prevent some of the problems described previously. We followed the human-centered design principles [49] with the goal of designing a system that is modeled in accordance with the characteristics, tasks, and requirements of the end users. However, in software engineering there are numerous methods for designing software applications [41,49] and using another design and evaluation method might therefore have led to different results.

### Recommendations for Future Research

Given the limited research efforts being invested to systematically involve the end users in the design of new teletreatments, the findings of this study (eg, the use of a decision matrix) could be applied in future telehealth projects. Sharing the experiences with tools for human-centered design processes will eventually lead to a better understanding of ways to develop user-friendly teletreatments, will enable comparison with products and the efficacy of different methods, and will ultimately lead to higher degrees of user acceptance for eHealth solutions. Mirror therapy has shown promising results in reducing phantom limb pain in 3 controlled studies, however, the evidence is still limited [7,8]. It is still not clear which patients may respond more favorably to mirror therapy than others, but at least some patients who experience no effect through mirror therapy could be more suitable for alternative methods such as virtual or augmented reality [50]. Compared with the mirror therapy approach, these treatment strategies are able to adapt the visual image to the perceived position and length of the phantom limb thereby making the visual illusion more vivid and real, which has been shown to be correlated with the effects of the treatment [6]. The results of our multicenter trial [10] will yield information about the potential effects of mirror therapy and the telerehabilitation platform in treating phantom limb pain in routine care, and will indicate further points for improvement of the platform. Within this trial we will also assess user acceptance of the service using a questionnaire based on the technology acceptance model [23,24].

### Conclusions

This study involved developing a mobile telerehabilitation platform for patients with phantom limb pain through an iterative user-centered design process. Our findings underline the importance of involving the users and other stakeholders in an iterative design process by our project, as well as the need for clear criteria to identify critical user requirements. The decision matrix presented here incorporates the views of various stakeholders and might help others systematically rate and prioritize user requirements. The reported findings and lessons learned might be of interest to health care providers, researchers, software designers, and other stakeholders when designing and evaluating new teletreatments. They may also potentially increase the likelihood of user acceptance of these applications.

## Appendix

Multimedia Appendix 1 Video demonstrating the use of the telerehabilitation platform.
